# Prevalence, antibiotic resistance, and genomic characterisation of *Campylobacter* spp. in retail chicken in Hanoi, Vietnam

**DOI:** 10.1099/mgen.0.001190

**Published:** 2024-01-31

**Authors:** Luu Quynh Huong, Thomas Chisnall, John D. Rodgers, Shaun A. Cawthraw, Roderick M. Card

**Affiliations:** ^1^​ National Institute of Veterinary Research (NIVR), 86 Truong Chinh Road, Dong Da district, Hanoi, Vietnam; ^2^​ Animal and Plant Health Agency, Woodham Lane, New Haw, Addlestone, UK

**Keywords:** antimicrobial susceptibility, *C. coli*, *C. jejuni*, *Campylobacter*, prevalence, Vietnam, whole genome sequencing

## Abstract

*Campylobacter* spp. are a leading cause of bacterial foodborne zoonosis worldwide, with poultry meat and products recognised as a significant source of human infection. In Vietnam there are few data regarding the occurrence, antimicrobial resistance, and genomic diversity of *Campylobacter* in poultry and poultry meat. The aim of this study was to estimate the prevalence of *Campylobacter* in chicken meat at retail in Hanoi, determine antimicrobial sensitivities of the *Campylobacter* isolated, and assess their genetic diversity. A total of 120 chicken meat samples were collected from eight traditional retail markets (*n*=80) and four supermarkets (*n*=40). *Campylobacter* was isolated following ISO 10272-1 : 2017 and identification verified by PCR. The prevalence of *Campylobacter* was 38.3 % (46/120) and *C. coli* was the most prevalent species in both retail markets (74 %) and supermarkets (88 %). The minimum inhibitory concentrations for ciprofloxacin, erythromycin, gentamicin, nalidixic acid, streptomycin, and tetracycline were determined by broth microdilution for 32 isolates. All characterised *Campylobacter* were resistant to ciprofloxacin, nalidixic acid, and tetracycline, with corresponding resistance determinants detected in the sequenced genomes. Most *C. coli* were multidrug resistant (24/28) and two harboured the erythromycin resistance gene *ermB* on a multiple drug-resistance genomic island, a potential mechanism for dissemination of resistance. The 32 isolates belonged to clonal complexes associated with both poultry and people, such as CC828 for *C. coli*. These results contribute to the One Health approach for addressing *Campylobacter* in Vietnam by providing detailed new insights into a main source of human infection and can inform the design of future surveillance approaches.

## Impact Statement

*Campylobacter* spp. are a major cause of foodborne bacterial disease and present a significant threat to public health globally with a considerable concomitant economic burden. *Campylobacter* are prevalent in livestock reared for food, and human infection is generally via handling or consumption of contaminated meat and meat products, such as chicken. In Vietnam consumption of poultry meat has almost doubled in the 5 years up to 2020. Given the lack of data on *Campylobacter* in Vietnam, we have investigated the occurrence and characteristics of *Campylobacter* present in chicken at retail in Hanoi. We show that the *Campylobacter* detected were of the same genotypes as those found in human infections in many countries. Furthermore, all characterised isolates were resistant to quinolone antibiotics, commonly used to treat human infection, and the majority were multidrug resistant (i.e. resistance to at least three classes of antibiotics). These findings reinforce the importance of good food hygiene and preparation measures to minimise exposure. This study has utilised standard methods for isolation, phenotypic characterisation and genomic typing providing data that is readily comparable across the One Health space and will inform risk assessments for Campylobacteriosis and dissemination of AMR both in Vietnam and in a global context.

## Data Summary

The whole genome sequences were deposited in the National Centre for Biotechnology Information (NCBI) National Library of Medicine under BioProject accession number PRJNA1016150.

## Introduction

*Campylobacter* spp., in particular *C. jejuni* and *C. coli*, are major causes of bacterial foodborne disease worldwide and are commonly present in food-producing animals. *Campylobacter* is responsible for 1.5 million cases of foodborne illnesses in the USA every year [1] and was the most reported zoonosis in the European Union in 2022 [2]. It has been estimated that *Campylobacter* spp. are responsible for 400–500 million cases of disease each year worldwide [3]. The number of foodborne illnesses and deaths caused by *Campylobacter* globally in 2010 has been estimated at over 95 million and >21 000, respectively [4]. Disease is typically a self-limiting diarrheal illness lasting 5 to 7 days, although immunocompromised and elderly patients have a higher risk for morbidity, mortality, and prolonged illness [5].

Handling and consumption of undercooked chicken meat or contaminated ready-to-eat food are common sources of infection [2]. *Campylobacter* is highly prevalent in poultry production systems, including broilers, layers, turkeys, and ducks [6]. *C. jejuni* colonises the chicken gut in high numbers, primarily in the caeca and small intestine, but it may also be found in liver and spleen [7]. Therefore, intestinal tracts of chickens are reservoirs of *Campylobacter* for on-farm transmission or cross-contamination of carcases during processing [8].

Worldwide, *C. jejuni* is responsible for 85 % of foodborne *Campylobacter* enteritis cases in humans and is also the most frequently isolated *Campylobacter* species from poultry, while the remaining cases are primarily attributed to *C. coli* [9]. There has been an increase in the prevalence of antimicrobial resistance in *Campylobacter* isolates over time and resistances to fluoroquinolones and macrolides are of particular concern, given their use for treatment of human infections [10, 11].

In Vietnam *Campylobacter* is recognised as an important cause of gastroenteritis [12] and has been detected in children and adults, with positive rates ranging from 2–20 % in patients presenting with diarrhoeal illness [13–15]. The consumption of chicken meat is regarded as the main source of human campylobacteriosis in Vietnam [12]. Consumption of poultry meat in Vietnam has almost doubled between 2015 and 2020 to 10 kg per capita [16], and ranks second to pork in meat consumption [17]. In a study from the Mekong delta, Vietnam, the prevalence of *Campylobacter* in chicken faeces was 32 % [18], while a study from the Can Tho Province reported a prevalence of 76 % [19]. In poultry carcases and food products *Campylobacter* contamination rates of between 15 and 41 % have been reported in Vietnam [17, 20–22]. Few publications have described the antimicrobial susceptibilities of Vietnamese *Campylobacter*, reviewed in [12], and these report a high occurrence of resistance to ciprofloxacin, nalidixic acid, and tetracycline [15, 18, 19, 21, 23]. A significant data gap is the absence of a detailed understanding of the genetic basis for antimicrobial resistance in *Campylobacter* from Vietnam, as published studies have focussed on generating susceptibility data only and none have published whole genome sequences. Genomic characterisation via multi-locus sequence typing indicate the diversity the *Campylobacter* population within an ecological space and has been successfully used to infer the expected origins of isolates via source attribution modelling [24] and the accuracy of attributions are improving with availability of AI and WGS data [25]. To date there is limited genomic information published that describes the population of *Campylobacter* contaminating chicken meat at retail in Vietnam [23], and such information is key to determine the extent to which chicken production and consumption is driving campylobacteriosis in people in Vietnam.

The aim of the present study was to assess the prevalence of *Campylobacter* in chicken meat at retail in Hanoi in 2019, and to determine the genome diversity and antimicrobial sensitivities of the strains isolated using Whole Genome Sequencing (WGS) and broth microdilution antimicrobial susceptibility testing.

## Methodology

### Isolation and identification of *Campylobacter*

A total of 120 chicken meat samples were collected from eight traditional retail markets (*n*=80) and four supermarkets (*n*=40) in Hanoi, Vietnam between March and May 2019. Each site was visited on four separate occasions for sample collection, and the time between visits was 2 weeks for the retail markets and 1–2 weeks for the supermarkets. Two chicken meat vendors were visited at four of the retail markets and three vendors were visited at the other four retail markets. One chicken carcass sample (approximately 300 g breast meat) was collected from each retail market vendor per visit, to ensure the samples did not come from the same chicken. At the supermarkets, one packaged chicken meat sample (approximately 500 g) was collected from each brand at each sampling visit, to ensure the samples did not come from the same chicken. At two supermarkets two different brands were collected, whereas at the other two supermarkets three different brands were collected.

The samples were processed immediately upon arrival at the laboratory of the National Institute of Veterinary Research following standard protocols for *Campylobacter* isolation and identification (ISO 10272-1 : 2017). Briefly, 10 g chicken breast meat without skin were homogenised in 90 ml of Preston broth (Oxoid, UK) using a stomacher (Seward, UK) for 1 min. Homogenates were incubated at 42 °C for 48 h in a microaerobic atmosphere generated in gas jars containing CampyGen sachets (Oxoid, UK). A 10 µl loopful of enriched broth was streaked onto modified cefoperazone charcoal deoxycholate agar (mCCDA; Oxoid, UK) and Preston agar (Oxoid, UK) and incubated at for 48 h as before. Colonies were examined by Gram-stain and wet mount for typical *Campylobacter* morphology and motility. After sub-culturing the isolates on Columbia blood agar with 5 % sterile defibrinated sheep blood (Oxoid, UK), oxidase, catalase, and hippurate tests were applied for further confirmation and speciation. Genus and species identification were verified by PCR using the primers in Table 1 on DNA extracts prepared from fresh plate cultures using the QIAamp DNA Blood Mini Ki (Qiagen, Germany) according to the kit protocol on a Biorad PCR System (BIO RAD). *Campylobacter* isolates, one colony per positive sample, were stored at −80 °C in 30 % (v:v) glycerol (Merck, Germany) in Brain Heart Infusion broth.

**Table 1.** PCR primers used to identify *Campylobacter* genus, *C. jejuni* and *C. coli*

Target species	Name of primer	Target gene	Sequence (5′−3′)	Product size (bp)	Reference
*C. jejuni*	MapAF	*MapA*	CTATTTTATTTTTGAGTGCTTGTG	589 bp	[66]
	MapAR		GCTTTATTTGCCATTTGTTTTATTA		
*C. coli*	Coli F	*ceuE*	AATTGAAAATTGCTCCAACTATG	462 bp	[66]
	Coli R		TGATTTTATTATTTGTAGCAGCG		
Campylobacter Genus	PL06	16S rRNA	GGTTAAGTCCCGCAACGAGCCGC	283 bp	[67]
	CAMPC5		GGCTGATCTACGATTACTAGCGAT		

The proportion of samples positive for *Campylobacter* and the 95 % confidence interval of the proportions were calculated from the multiple of estimated standard error and an approximately normal sampling distribution [26]. The Chi-squared test with one degree of freedom [26] tested the null hypothesis that there was no significant difference between the proportion of *Campylobacter* positive samples in meat from supermarkets and meat from retail markets.

*Campylobacter* strains were recovered from frozen storage by culture on Columbia blood agar (Oxoid, UK) and shipped to the UK on charcoal transport swabs, according to IATA regulations, for additional testing (see below). In the UK, *Campylobacter* isolates were cultured in a microaerobic environment (5–10 % CO_2_, 5–7 % O_2_) on mCCDA and 7 % sheep blood agar with 0.1 % actidione plates to ensure purity prior to storage. For speciation, ethanol/formic acid extracts of colonies were analysed by mass spectrometry (Bruker Microflex MALDI-TOF MS, Bruker Daltonics Ltd, UK) and Bruker MBT Compass software (version 4.1.70).

### Antimicrobial susceptibility testing

The minimum inhibitory concentrations (MIC) for six antimicrobials (ciprofloxacin, erythromycin, gentamicin, nalidixic acid, streptomycin, and tetracycline) were determined by broth microdilution using Thermofisher Sensititre EUCAMP2 plates according to the manufacturer’s protocol. Plates were incubated microaerobically at 41 °C for 24 h. MICs were interpreted using the European Committee on Antimicrobial Susceptibility Testing (EUCAST) epidemiological cut-off (ECOFF) values as wild-type or non-wild-type [27]. ECOFFs distinguish microorganisms without (wild-type) and with phenotypically detectable acquired resistance mechanisms (non-wild-type) to the antimicrobial in question (https://mic.eucast.org/). Isolates with non-wild-type susceptibilities have been termed microbiologically ‘resistant’, although it is recognised this is not necessarily synonymous with clinical resistance. Multidrug resistance was defined as resistance to three or more antimicrobial classes [27]. In the panel of antimicrobials tested the quinolone class is represented by ciprofloxacin and nalidixic acid.

### Whole genome sequencing

*Campylobacter* isolates were suspended in 0.1 M phosphate buffered saline (pH7.2) (PBS) to a turbidity of 0.5 McFarland and centrifuged to produce pellets. Supernatants were removed and pellets re-suspended in PBS prior to extraction and purification of DNA using a semi-automated extraction protocol entailing MagMAX CORE extraction kits and the KingFisher Flex system (both Thermo Fisher Scientific, Basingstoke, UK). Extracted DNA was processed for WGS at APHA Central Sequencing Unit (APHA Weybridge, UK). Libraries were prepared with a Nextera XT DNA sample preparation kit (Illumina, San Diego, CA), according to the manufacturer’s instructions. WGS was carried out using the Illumina NextSeq platform (Illumina Inc., San Diego, California, USA) for short read sequencing. Raw sequences were analysed using the Nullabor two pipeline [28], using as reference the published genome of *Campylobacter coli* strain 15–537360 [29], SPADES for genome assembly (version 3.14.1 [30]) and Prokka for annotation (version 1.14.6 [31]). The presence of genes and point mutations conferring antimicrobial resistance (AMR) was assessed using AMRFinderPlus [32]. The sequence type (ST) was determined with MLST (version 2.19.0; https://github.com/tseemann/mlst) using the pubMLST database [33]. New alleles and STs were submitted to the *Campylobacter jejuni/coli* database at pubMLST. The core genome SNP alignment for *C. coli* isolates was produced by Snippy (version 4.6.0; https://github.com/tseemann/snippy) and used to build a bootstrapped (*n*=200) phylogenetic tree with RAxML [34] and annotated using iTOLv3 [35].

## Results and discussion

### Prevalence of *Campylobacter* in chicken meat

The overall prevalence of *Campylobacter* in chicken meat was 38.3 % (46/120 samples; Table 2). The proportion of positive samples from retail markets was significantly higher (Chi^2^=13.7, *P*<0.001) than supermarkets, 47.5 % (*n*=80) and 20 % (*n*=40) respectively. *Campylobacter* were found at all of the visited retail markets and supermarkets. *C. coli* was the most prevalent species at both market types: 28/38 (74 %) positive samples from retail markets and 7/8 (88 %) positive samples from supermarkets. A similar predominance of *C. coli* was also observed in a survey of fresh chicken carcasses conducted in Hanoi [17]. The difference in prevalence between retail markets and supermarkets is likely due to one or more factors such as source and types of poultry, slaughter, preparation, storage and hygiene practices, and warrants further study. Poultry at retail markets is generally slaughtered by retailers at point of sale, or at home by consumers, whereas chicken meat in supermarkets has been prepared at slaughterhouses owned or contracted by large producers [16]. Hygiene standards in slaughterhouses are regarded as superior to those in retail markets and can include practices such as sampling to monitor for pathogens [16]. Differences in hygiene standards may have contributed to the increased prevalence of *Campylobacter* in chicken meat at retail markets. Furthermore, in Vietnam the poultry production and distribution networks for white (or exotic) broilers and coloured and spent hens differ, with the latter sold mainly through retail markets [16]. These differences may affect the prevalence and species of *Campylobacter* detected and warrant further investigation. Another study in Vietnam reported a moderately higher prevalence (although not significant) in supermarkets (57 %) compared to traditional markets (47 %) [17]. The increased prevalence in supermarket chicken reported by [17] relative to the current study may be related to methodological differences. Enrichment in Bolton broth as used by [17] may have increased sensitivity for detection of stressed and low numbers of *Campylobacter* relative to Preston broth which was used in this study (ISO 10272-1 : 2017).

**Table 2.** Prevalence of *Campylobacter* at retail markets and supermarkets in Hanoi, Vietnam

Sample origin	Samples tested	*C. jejuni* positive samples	*C. coli* positive samples	Total positive samples	Prevalence	Confidence interval (95 %)
Retail market	80	10	28	38	47.5 %	36.6–58.4 %
Supermarket	40	1	7	8	20.0 %	7.6–32.4 %
Total	120	11	35	46	38.3 %	29.6–47.0 %

A prevalence of 38.3 % is similar to that found in earlier studies undertaken in Vietnam, which showed 31 % (from 100 samples tested) *Campylobacter* contamination in chicken meat from retail markets in Hanoi [22], 28.3 % (from 60 samples tested) in poultry meat collected from hospitals, schools and factory canteens [20], and 41.1 % (from 107 samples tested) in fresh chicken carcasses [17]. Interestingly, it is considerably lower than rates seen in chicken meat samples in many other countries, including: Cambodia (80.9 %, 139 samples tested [36]), Japan (64.7  %, 170 samples tested [37]), Poland (70 %, 70 samples tested [38]), New Zealand (69.7 %, 175 samples tested [39]), and Northern Ireland (91 %, 336 samples tested [40]). The lower rate in the Hanoi markets investigated is notable but care must be taken in drawing comparisons, as sampling strategies and numbers of samples taken vary considerably. For example, studies in Italy with localized sampling reported prevalences in fresh poultry meat and ready-to-cook products in retail outlets that varied from 17–86 % [41–44]. Furthermore, skin typically accumulates more contamination than the actual meat [45] and our study tested skinless chicken breast meat whilst other studies included samples with skin.

### Diversity of *Campylobacter*

**Fig. 1.**
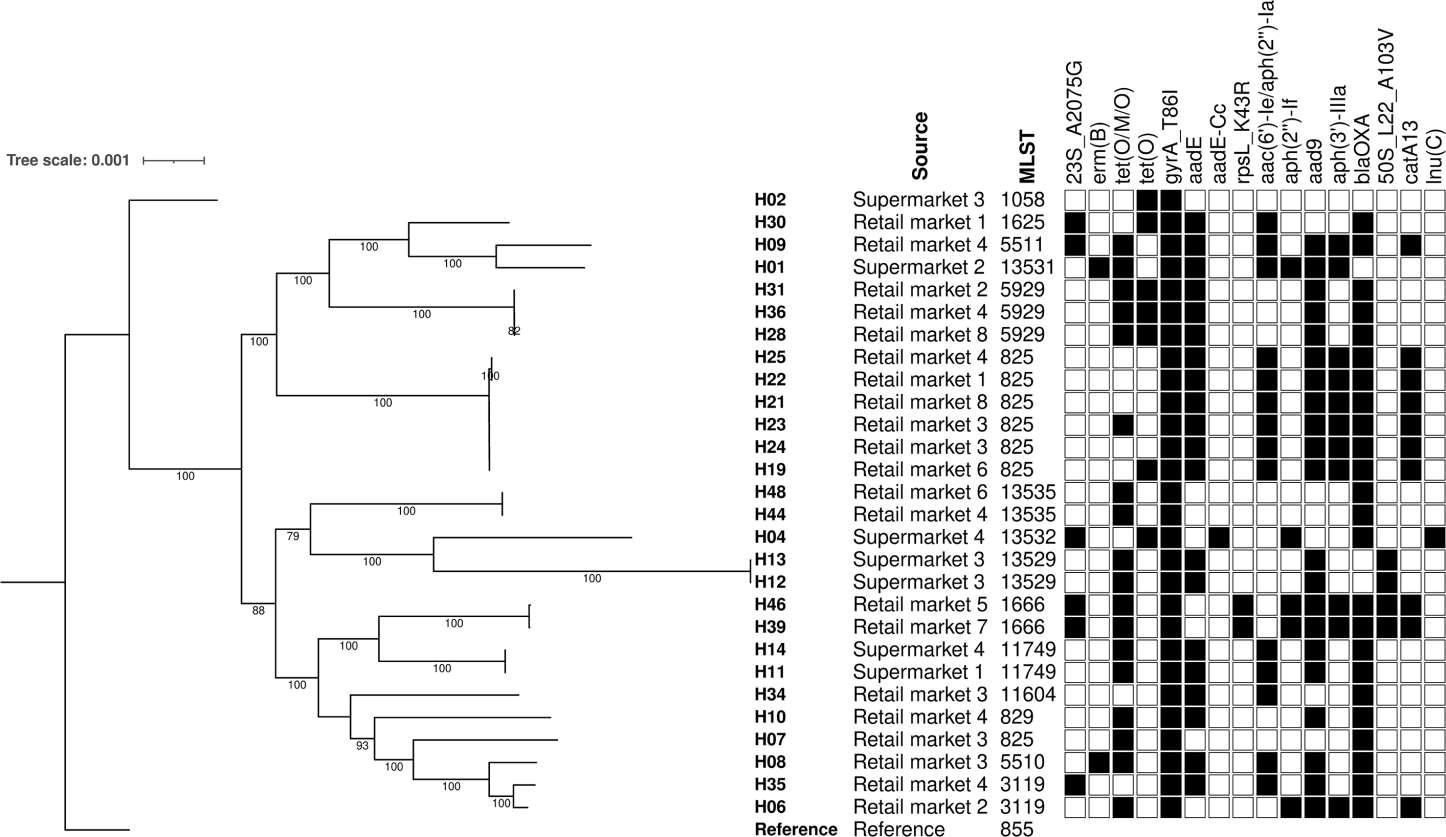
Maximum-likelihood phylogenetic tree generated from core genome single-nucleotide polymorphisms of *C. coli* isolates. Genetic relatedness is indicated by branch length and position confidence in clades by bootstrap confidence values >80 % are displayed on the tree. Also shown are the source, multi-locus sequence type, and carriage of AMR genes and point mutations (indicated by black square). Reference was *C. coli* strain 15–537360.

Only 32 of the 46 isolates shipped to the UK were still viable upon receipt. These were all identified by MALDI-ToF as *Campylobacter*; 28 *C*. *coli* and four *C*. *jejuni* (Table S1, available in the online version of this article), which matched the PCR speciation data. The *C. coli* isolates comprised 15 sequence types, of which four were newly identified in this study (ST13529, ST13531, ST15532, and ST13535; Table S2). All 11 previously described STs and three new STs were from the ST-828 clonal complex (Tables S1 and S2). CC828 has been defined as the main *C. coli* CC following an analysis of 11 920 *C*. *coli* genomes in public repositories and proposed as a ‘chicken infection specialist’ [46], although others have hypothesised that CC828 is adapted to a generalist lifestyle [47]. CC828 has been the most reported CC in studies of human *C. coli* infections in China [46] and in the UK [48], and has been described in poultry-derived samples from Vietnam [18, 23]. There was considerable diversity in the core SNP genome phylogenetic tree of the *C. coli* isolates and within this limited sample size no ST was detected that was common to both retail market and supermarket samples (Fig. 1). In this study 87.5 % of the *Campylobacter* isolates from chicken meat sample originating from intensive production systems were characterised as *C. coli,* whilst a survey of Vietnamese chicken flocks that included small (backyard), medium and large-scale farming systems reported a similar proportion of *C. coli and C. jejuni* [18]. *Campylobacter coli* belonging to CC828 is recognised as a lineage that has evolved alongside the development of intensive agriculture systems [47].

The four *C. jejuni* isolates were ST464 (CC464), ST535 (two isolates; CC460), and ST12630 (CC354) (Table S1). In the pubMLST database (accessed 23 August 2023) ST12630 has been reported previously only from human samples in Vietnam collected in 2015. Similarly, CC460 has been reported in chicken, duck, and pig samples from Vietnam [18, 23]. Analysis of c. 39 800 publicly available *C. jejuni* and *C. coli* genomes from around the world determined clonal complexes 354, 460 and 464 are strongly associated with samples from the chicken meat production system [49]. Furthermore, in a UK study these complexes were found to be associated with ciprofloxacin resistance and human infection [48], and a link with human infections was also noted in Polish, Australian, Finnish, and Chinese studies [46, 50–52].

### Antimicrobial resistance

Antimicrobial susceptibilities were determined for six antibiotics by broth microdilution assay and MICs interpreted using EUCAST ECOFF values. The 32 isolates were all resistant to ciprofloxacin, nalidixic acid, and tetracycline. All four *C. jejuni* isolates were susceptible to the remaining three antimicrobials (streptomycin, gentamicin, and erythromycin) and none were multidrug resistant, a resistance pattern commonly reported for *C. jejuni* in the European Union and the United Kingdom [10] and Vietnam [21, 23]. For *C. coli* (*n*=28) additional resistances were detected in many isolates: streptomycin (*n*=23), gentamicin (*n*=18), and erythromycin (*n*=8) (Table S1). Multidrug resistance was common amongst the *C. coli* isolates, with 24 out of 28 isolates (86 %) having resistance to three or more antimicrobial classes, a striking proportion relative to statistics for *C. coli* isolated from broiler flocks in EU member states in 2019/20 where just 3.9 % were reported as multidrug resistant, using the same antimicrobial panel as used in this study [53]. The relative abundance of *C. coli* in these samples coupled with the predilection of this species to acquire resistance elements within its genome highlight the need for tight controls on release of antimicrobials into the environment via food producing animals and medical treatment [54].

The genetic basis for antimicrobial resistance was assessed by WGS analysis (Fig. 1), and there was good correspondence between AMR phenotype and genotype (Table S1). Resistance to quinolones was associated with the mutation in *gyrA* giving rise to the single step T86I amino acid change, commonly associated with quinolone resistance in *Campylobacter* [55]. Tetracycline resistance was most commonly associated with *tet*(O/M/O) encoding a ribosomal protection mosaic protein, which has been reported in *Campylobacter* from several sources [56]. The *tet*(O) gene was present in five *C. coli* isolates. Gentamicin resistant isolates harboured either *aac(6')-Ie/aph(2'')-Ia* or *aph(2'')-If*; one resistant isolate harboured both genes; *aph (2'')-If* also confers resistance to kanamycin [57] but susceptibility to this antibiotic was not tested. Twenty-three *C. coli* isolates were resistant to streptomycin, with each harbouring a corresponding resistance determinant: *aadE* (*n*=20), *aadE-Cc* (*n*=1) or a K43R mutation in *rpsL* (*n*=2). Additional AMR genes were detected by WGS but the corresponding resistance phenotype was not tested: *aadA9* (spectinomycin) *n*=21; *aph(3')-IIIa* (amikacin / kanamycin) *n*=11; *bla*_OXA_ (beta-lactam) *n*=27 [of which 19 were *bla*_OXA-193_]; *catA13* (chloramphenicol) *n*=10; and *lnu(C*) (lincomycin) *n*=1 (Table S1). Many of the *C. coli* isolates characterised in this study had multiple genes associated with aminoglycoside resistance, encoding for aminoglycoside-modifying enzymes; aminoglycoside adenyltransferase (AAD), aminoglycoside phosphotransferase (APH) and aminoglycoside acetyltransferase (AAC). This portfolio of genes may be reflective of a genomic resistance island for aminoglycosides as previously described for *C. coli* (sequence types, ST5604, ST1586 and ST1625) recovered from chickens in China [58]. In this study a single ST1625 isolate was identified however further analyses are required to confirm the location and co-location of aminoglycoside resistances on all the isolates to confirm the presence of a genomic resistance island.

**Fig. 2.**
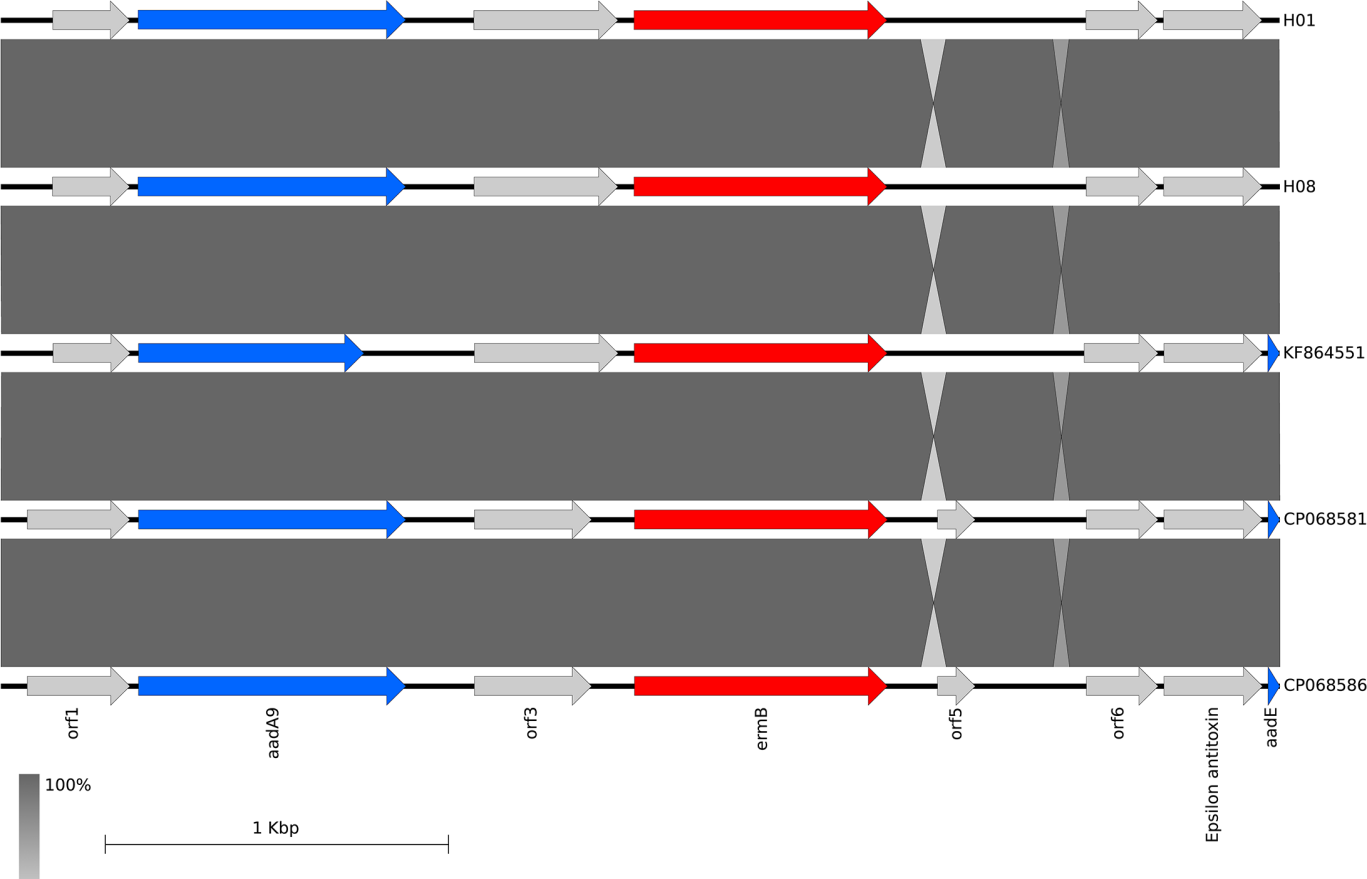
Genomic synteny comparisons of the *ermB* region of selected genomes. Isolates H01 and H08 are compared to the corresponding region in the exemplar genomes of isolates *C. jejuni* KF864551 [68] and *C. coli* CP068581 and CP068586 [69]. Antimicrobial resistance genes are shaded in red (*ermb*) or blue (*aadA9* and *aadE*); other coding sequences are shaded in light grey and described in Table S3. Regions of sequence similarity between isolates are shown by grey shading. Image generated using Easyfig [64] and the BLASTn algorithm [61].

Erythromycin resistance was associated with an A2075G point mutation in the 23S rRNA gene in six isolates and with *ermB* in two isolates (isolate H01 from a supermarket and H08 from a retail market) (Fig. 1). The presence of the A2075G chromosomal mutation in five different STs is an important finding as it may indicate a wide distribution and possibly relates to a widespread usage of erythromycin in poultry in Vietnam [59]. Furthermore, the mutation was not associated with a fitness cost in *C. coli* [60] and so could persist in the absence of antibiotic selection. Isolates H01 and H08 were resistant to all six antimicrobials tested. In addition to *ermB* they carried the T86I *gyrA* variant and five further AMR genes: *tet*(O/M/O), *aadE*, *aad9*, *aac(6')-Ie/aph(2'')-Ia*, and *bla*_OXA-193_; H01 additionally harboured *aph(3')-IIIa* and *aph(2'')-If* (Fig. 1). The *ermB* gene was located on a small contig in the WGS from both isolates (H01 3726 bp; H08 3725 bp) which also carried the aminoglycoside resistance gene *aadA9* and four additional predicted coding sequences (detailed in Table S3) (Fig. 2). In blastn analysis [61] the contigs had 100 % nucleotide identity with each other and >99.9 % nucleotide identity with sequences of a multiple drug-resistant genomic island previously described in *C. coli* and *C. jejuni*. These genomic islands have been classified into at least eleven types [62, 63] and the contigs from this study had the highest nucleotide identity with Type VII. However, the contigs did not span the entire island precluding definitive assignment. The two contigs were compared using EasyFig [64] on the corresponding region of three isolates identified by Blast, including KF864551 the Type VII exemplar [62], revealing high sequence identity and conserved gene synteny (Fig. 2).

The two isolates with *ermB* were of different STs and divergent in the core SNP genome phylogenetic tree (Fig. 1), providing the first evidence that a multiple drug-resistant genomic island is present in different lineages of *C. coli* in Vietnam. This is significant as *ermB* in *C. coli* confers resistance to erythromycin and elevated resistance to other macrolides including azithromycin [65], and together with other resistances, particularly to quinolones, significantly restricts therapeutic options for treatment of human infections.

This study has provided detailed insight into the prevalence of *Campylobacter* in retail chicken in Hanoi, Vietnam, and the antimicrobial resistances present, which can inform risk assessments for Campylobacteriosis and dissemination of AMR. Although the prevalence reported here is low in comparison to many other countries, consumers should continue to take measures to minimise exposure. These can include chilling food, cooking chicken correctly, avoiding cross-contamination and ensuring good personal hygiene. The *Campylobacter* isolates examined by WGS were from clonal complexes associated with poultry and human infection, highlighting the potential risk for consumers. The study has provided the first detailed insight into the genetic basis of antimicrobial resistance in *Campylobacter* in Vietnam, thereby addressing an important evidence gap. Although most human infections of *Campylobacter* are self-limiting, the high occurrence of quinolone resistance (100 %) and erythromycin resistance (25 %) in these isolates warrants consideration when considering effective treatment of human infections. The predominance of multidrug resistant *C. coli* contamination on meat for human consumption highlighted in this study emphasises the need for an expanded programme of surveillance, susceptibility testing, and genomic characterisation of *Campylobacter* in poultry at retail to evaluate the significance of this risk in the context of One Health and the global campaign to control AMR. Employing similar approaches, including whole genome sequencing, for human clinical isolates will help inform the One Health approach to addressing *Campylobacter* and contribute to source tracing.

## Supplementary Data

Supplementary material 1
